# Genome-Wide Analysis of the R2R3-MYB Gene Family in *Fragaria* × *ananassa* and Its Function Identification During Anthocyanins Biosynthesis in Pink-Flowered Strawberry

**DOI:** 10.3389/fpls.2021.702160

**Published:** 2021-08-30

**Authors:** Jiaxin Liu, Jian Wang, Mingqian Wang, Jun Zhao, Yang Zheng, Tian Zhang, Li Xue, Jiajun Lei

**Affiliations:** ^1^College of Horticulture, Shenyang Agricultural University, Shenyang, China; ^2^Genepioneer Biotechnologies Co. Ltd, Nanjing, China

**Keywords:** *Fragaria* × *ananassa*, R2R3-MYB genes, genome-wide analysis, pink flower, anthocyanins biosynthesis

## Abstract

The strawberry (*Fragaria* × *ananassa*) is an economically important fruit throughout the world. The large *R2R3-MYB* gene family participates in a variety of plant functions, including anthocyanin biosynthesis. The present study is the first genome-wide analysis of the *MYB* gene family in the octoploid strawberry and describes the identification and characterization of the family members using the recently sequenced *F.* × *ananassa* genome. Specifically, we aimed to identify the key MYBs involved in petal coloration in the pink-flowered strawberry, which increases its ornamental value. A comprehensive, genome-wide analysis of *F.* × *ananassa R2R3-FaMYBs* was performed, investigating gene structures, phylogenic relationships, promoter regions, chromosomal locations, and collinearity. A total of 393 *R2R3-FaMYB* genes were identified in the *F.* × *ananassa* genome and divided into 36 subgroups based on phylogenetic analysis. Most genes with similar functions in the same subgroup exhibited similar exon-intron structures and motif compositions. These *R2R3-FaMYBs* were unevenly distributed over 28 chromosomes. The expansion of the *R2R3-FaMYB* gene family in the *F.* × *ananassa* genome was found to be caused mainly by segmental duplication. The Ka/Ks analysis indicated that duplicated *R2R3-FaMYBs* mostly experienced purifying selection and showed limited functional divergence after the duplication events. To elucidate which *R2R3-FaMYB* genes were associated with anthocyanin biosynthesis in the petals of the pink-flowered strawberry, we compared transcriptional changes in different flower developmental stages using RNA-seq. There were 131 differentially expressed *R2R3-FaMYB* genes identified in the petals, of which three genes, *FaMYB28*, *FaMYB54*, and *FaMYB576*, appeared likely, based on the phylogenetic analysis, to regulate anthocyanin biosynthesis. The qRT-PCR showed that these three genes were more highly expressed in petals than in other tissues (fruit, leaf, petiole and stolon) and their expressions were higher in red compared to pink and white petals. These results facilitate the clarification on the roles of the *R2R3-FaMYB* genes in petal coloration in the pink-flowered strawberry. This work provides useful information for further functional analysis on the *R2R3-FaMYB* gene family in *F.* × *ananassa*.

## Introduction

The cultivated strawberry (*Fragaria* × *ananassa* Duch.) is an important horticultural crop throughout the world. It is beneficial to human health due to its high nutrient and flavonoid content and is attractive to consumers because of its sweet flavor and rich red coloration (Ertan et al., 2020). The appearance of the pink-flowered strawberry, which derived from intergeneric hybridization (*Fragaria* × *Potentilla*) has increased its ornamental value (Xue et al., 2019). It can be used for landscaping and flourishes as a potted plant for ornamental. In the strawberry, anthocyanins are the main secondary metabolites responsible for the coloration of petals and fruits (Xue et al., 2016). The regulation of the anthocyanin biosynthetic pathway has been widely established in plants (Gu et al., 2019). Three transcription factor families (MYB, bHLH, and WD40) are involved in the regulation of anthocyanin biosynthesis; of these, MYB transcription factors play key roles (Allan et al., 2007; Jaakola, 2013; Xu et al., 2015). Three new members of the R2R3-MYB family (*PhASR1*, *PhASR2*, and *PhASR3*) that induce anthocyanin synthesis have been identified in *Petunia*, which interact with the transcription factors *PhAN1* and *PhAN11* to participate in the formation of the anthocyanin regulatory complex (Zhang et al., 2019). In the apple, *MdMYB1* was a pivotal regulator of anthocyanin biosynthesis, specifically regulating anthocyanin in the pericarp (Ban et al., 2007; Zhu et al., 2011). To date, several MYBs related to fruit coloration in *F.* × *ananassa* have been identified, including *FaMYB1*, *FaMYB5*, *FaMYB9*, *FaMYB10*, and *FaMYB11*, of which *FaMYB10* was found to be a key gene regulating anthocyanin synthesis in the strawberry fruit (Aharoni et al., 2001; Schaart et al., 2013; Medina-Puche et al., 2014; Wang et al., 2020; Zhang et al., 2020). However, how the *MYB* genes regulate the petal coloration of the pink-flowered strawberry is still unknown.

MYB transcription factors form one of the largest transcription regulatory families in plants (Martin and Paz-Ares, 1997; Liu et al., 2015). Many studies have confirmed that MYB transcription factors are involved in a variety of biological processes in plants, including growth and development, stress resistance, and secondary metabolism (Zhang et al., 2012; Jiang et al., 2018; Rahim et al., 2019). MYB proteins contain highly conserved repeats in the N-terminal DNA-binding domain and a variety of C-terminal regulatory regions responsible for gene regulation. The MYB domain is typically composed of 1–4 imperfect amino acid repeats (R), which consist of approximately 52 amino acids. Based on the number of conserved amino acid sequences, the MYB family can be differentiated four subclasses, 1R-MYBs, R2R3-MYBs, R1R2R3-MYBs, and 4R-MYBs, of which the R2R3-MYBs are the most common MYB transcription factors in plants (Stracke et al., 2001). R2R3-MYBs contain two adjacent repeats (R2 and R3) and form a modular structure with an N-terminal DNA binding domain and an activation or inhibition domain usually located at the C-terminal. And it has been divided into 25 subgroups in *Arabidopsis*, according to the conserved DNA binding domain and amino acid motifs in the C terminal domains (Dubos et al., 2010). The regulatory role of several R2R3-MYBs in flavonoid synthesis has been identified in *Arabidopsis* where *AtMYB3*, *AtMYB4*, *AtMYB7*, and *AtMYB32* act as transcriptional repressors regulating anthocyanin biosynthesis in subgroup 4, while *AtMYB75*, *AtMYB90*, *AtMYB113*, and *AtMYB114* are transcriptional promoters in subgroup 6 (Dubos et al., 2010). Previous studies have conducted extensive genome-wide analyses of MYB transcription factors in various plants, including *Arabidopsis* (Dubos et al., 2010), rice (Smita et al., 2015), *Manihot esculenta* (Ruan et al., 2017), *Gossypium hirsutum* (Salih et al., 2016), and Chinese pear (Cao et al., 2016). By determining homology with *Arabidopsis* genes, it is possible to preliminarily predict the gene functions of *MYB* genes in other species, which is useful for further elucidating the role of the *R2R3-MYB* genes in anthocyanin biosynthesis in the pink-flowered strawberry.

The octoploid strawberry genome is one of the most complex plant genomes. It stems from four different diploid ancestors and is an allopolyploid. The recently published genome sequence of the octoploid strawberry provides a strong tool for the identification, analysis, and utilization of the entire *FaMYB* gene family (Edger et al., 2019). At present, 120 *MYB* gene family members have been identified in *F. vesca* L., which may contain 105 *R2R3-MYB* genes (Yuan et al., 2019). However, there is little information regarding the MYB superfamily in the octoploid strawberry. How did the MYB genes evolve from a diploid strawberry genome to an octoploid genome? Are there any differences in the genes regulating the coloration of the strawberry fruit and pink petals? In the present study, we conducted a genome-wide analysis of the *R2R3-MYB* genes in the octoploid strawberry, including physicochemical properties, gene structure, promoter characteristics, chromosome location, and collinear relationships. Furthermore, three *R2R3-FaMYB*s were identified, which may play important roles in anthocyanin biosynthesis in the pink-flowered strawberry. These findings will provide a basis for the characterization of novel *R2R3-MYB*s involved in anthocyanin biosynthesis and help to improve the quality of strawberry fruits and flowers. Our genome-wide analysis will assist further exploration of the functional characteristics of *R2R3-FaMYB* proteins in the octoploid strawberry.

## Materials and Methods

### Genome-Wide Identification of *R2R3-FaMYB* Genes in the Octoploid Strawberry

The octoploid strawberry (*F.* × *ananassa*) genomic data file stem was downloaded from the GDR database.^1^ The corresponding MYB protein sequences were downloaded from the *Arabidopsis* database (TAIR; http://www.Arabidopsis.org/). The candidate R2R3-type MYB members in the octoploid strawberry were identified by a local BLASTP search with *Arabidopsis*, then all MYB-containing sequences in strawberry were further investigated using the hidden Markov model of the Myb-DNA-binding domain (PF00249) to search against the octoploid strawberry genome to identify candidates with *E*-value<1e-10. The gene structures of R2R3-type *MYB* genes were analyzed according to the GFF annotation file of the gene position information in the octoploid strawberry database. All sequences were further investigated using different tools, including the NCBI-Conserved Domains Database (CDD) web server, Pfamscan, and SMART. The presence of the conserved R2R3-MYB motif in proteins was determined using the MEME Suite 5.3.0 with the parameters of arbitrary repetition, maximum motif number of 1–15, and optimal motif width of 6–250 amino acids (Bailey et al., 2009).

### Phylogenetic Analysis

The R2R3-MYB protein sequences of the octoploid strawberry and Arabidopsis were used to generate phylogenetic trees *via* MAFFT v7.427 multiple sequence alignments with the default parameters. A maximum likelihood (ML) phylogenetic tree was constructed using MEGA 7.0 software with a bootstrap value of 1,000 and visualized using FastTree software, in which JTT was the best substitution model. Additionally, a separate phylogenetic tree with all the R2R3-FaMYB protein sequences in the octoploid strawberry was constructed using the same methods for further analysis.

### Analysis of the Gene Structure and Promoter Characteristics of *R2R3-FaMYB*s

The gene structures of all *R2R3-FaMYB* genes were displayed by the TBtoolsV0.67 software using the genomic sequences and coding regions of the *R2R3-FaMYB* genes, including exon and intron numbers and lengths. The *R2R3-FaMYB* promoter regions of 2000bp regions upstream of the translational start sites ATG were examined based on their positions in the octoploid strawberry genome, which was used to identify the cis-elements in the promoters according to the Plant CARE database.^2^

### *R2R3-FaMYBs* Physical Localization, Collinearity Analysis, and Ka/Ks Calculation of Duplicated *R2R3-FaMYB* Genes

The genome annotation data was collected and mapped on the chromosomes using the MapChart 2.3 software to identify the physical chromosomal location of all *R2R3-FaMYB* genes. The collinearity of intraspecific and interspecific genes was determined using the Multiple Collinearity Scan toolkit (MCSscanX, gap_penalty: −1, *E*-value: 1e-10) and visualized by using the Circos multiple synteny plot, in which the interspecies were *F. vesca* and *Rosa chinensis*. To further estimate duplication events, the non-synonymous rate (Ka), synonymous rate (Ks), and evolutionary constraint (Ka/Ks) between the duplicated pairs of *R2R3-FaMYBs* were calculated using TBtools V0.67 (Chen et al., 2020).

### Transcriptome Data Analysis

For the identification of flower color-related *R2R3-FaMYB* genes in the pink-flowered strawberry, we used our previously reported RNA-seq data from three flower developmental stages including bud stage (L), beginning coloration stage (Z), and big bud stage (D; Xue et al., 2019). The RNA-seq data were re-analyzed in accordance with the octoploid strawberry genomic data. The raw data were removed the reads that contained adaptor contamination, low quality bases and undetermined bases by using fastp software with default parameter. The software HISAT2 was used to map reads to the octoploid strawberry genome, then the mapped reads were assembled using StringTie with default parameters.^3^ A comprehensive transcriptome was reconstructed *via* gffcompare by merging all transcriptomes from all samples, and then the expression levels of all transcripts (FPKM value) were estimated by StringTie. The differentially expressed mRNAs were selected with fold change>2 or fold change<0.5 and with parametric F-test comparing nested linear models (*p*<0.05) by R package edgeR.

### Plant Materials, RNA Extraction, and qRT-PCR Analysis

**Figure 1:**
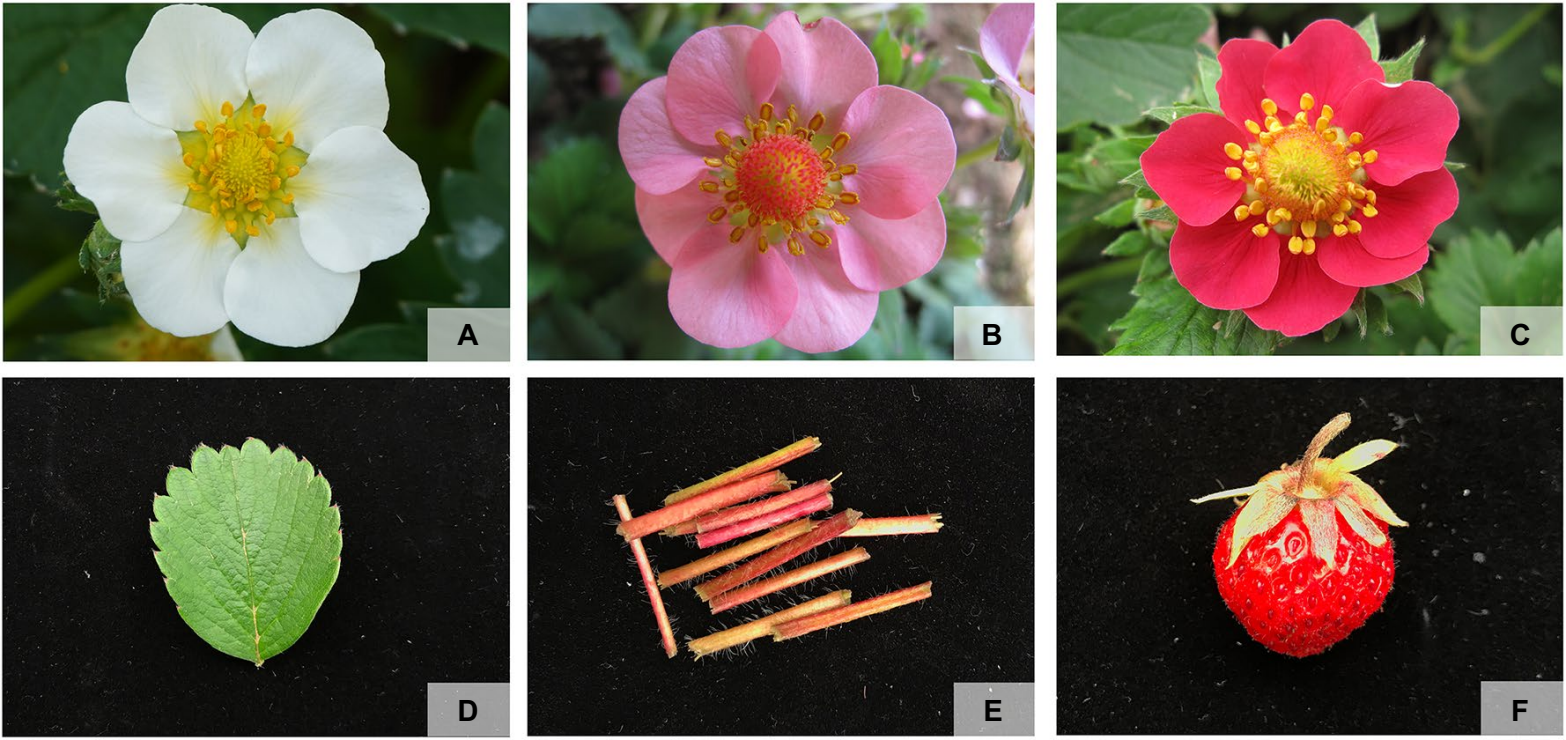
The materials used to detect the *R2R3-FaMYB* gene expression. **(A)** Fresh petals with white color at the full-bloom stage, **(B)** fresh petals with pink color at the full-bloom stage, **(C)** fresh petals with red color at the full-bloom stage, **(D)** leaf, **(E)** petiole, **(F)** fruit.

The pink-flowered strawberry was provided by the Shenyang Agricultural University, Liaoning, China. To detect the *R2R3-FaMYB* gene expression, three different fresh petals with red, pink, and white color at the full-bloom stage were sampled from the cross of Pink Princess × Pretty Beauty (Xue et al., 2019). And different tissues consisting of petal, leaf and petiole were sampled from the cultivar “Sijihong” (Figure 1). In order to select genes related to petal coloration, a phylogenetic tree was constructed using MEGA7.0 showing the different *R2R3-FaMYB* genes and other known anthocyanin synthesis related *R2R3-MYBs*, such as *AtMYBPAP1*, *MdMYB10*, *MdMYB110a*, *FaMYB1*, *FaMYB10*, and *AN2*. The reliability of the predicted tree was tested using bootstrapping with 1,000 replicates and LG+G model. All samples were immediately frozen in liquid nitrogen and stored at −80°C for RNA extraction. Total RNA was extracted using a modified CTAB method. The quality and concentration of each RNA sample were determined using gel electrophoresis and a NanoDrop 2000 spectrophotometer (Thermo Scientific, Waltham, MA, United States). The qRT-PCR experiments were conducted to analyze expression levels of *R2R3-FaMYB* genes related to anthocyanin biosynthesis among the different flower colors and tissues. The Applied Biosystems 7500 Real-Time PCR system (Applied Biosystems, Waltham, MA, United States) was used for the qRT-PCR reactions with the ChamQTM Universal SYBR® qPCR Master Mix (Vazyme, Nanjing, China) to amplify a final volume of 20μl. All qRT-PCR reactions were carried out with three independent biological replicates. The internal reference was the strawberry *FaDBP* gene (Schaart et al., 2002). All the data were subjected to statistical analysis using Duncan’s multiple range test (SPSS ver.17.0). The specific *R2R3-FaMYB* primers were designed by Primer 3.0 software and listed in Supplementary Table S1.

## Results

### Identification and Characteristics of Sequenced Octoploid Strawberry *R2R3-MYB* Family Genes

The MYB family protein sequences of *Arabidopsis* were downloaded and used as seed sequences to search the octoploid strawberry database to identify homologous *R2R3-MYB* genes in *F.* × *ananassa*. A total of 737 *FaMYB* genes were identified, accounting for about 0.6819% of all annotated sequences in the octoploid strawberry genome. Four different subfamilies were differentiated based on the number and location of domain repeats; these included 321 *1R-MYB* genes, 393 *R2R3-MYB* genes, 17 *R1R2R3-MYB* genes, and *6 R1R2R3R4-MYB* genes. Among them, the *R2R3-MYBs* (393 genes) were the largest *MYB* subgroup, comprising 53.32% of *FaMYB* genes.

### Phylogenetic Analysis of *R2R3-MYB* Genes in *F.* × *ananassa*

**Figure 2:**
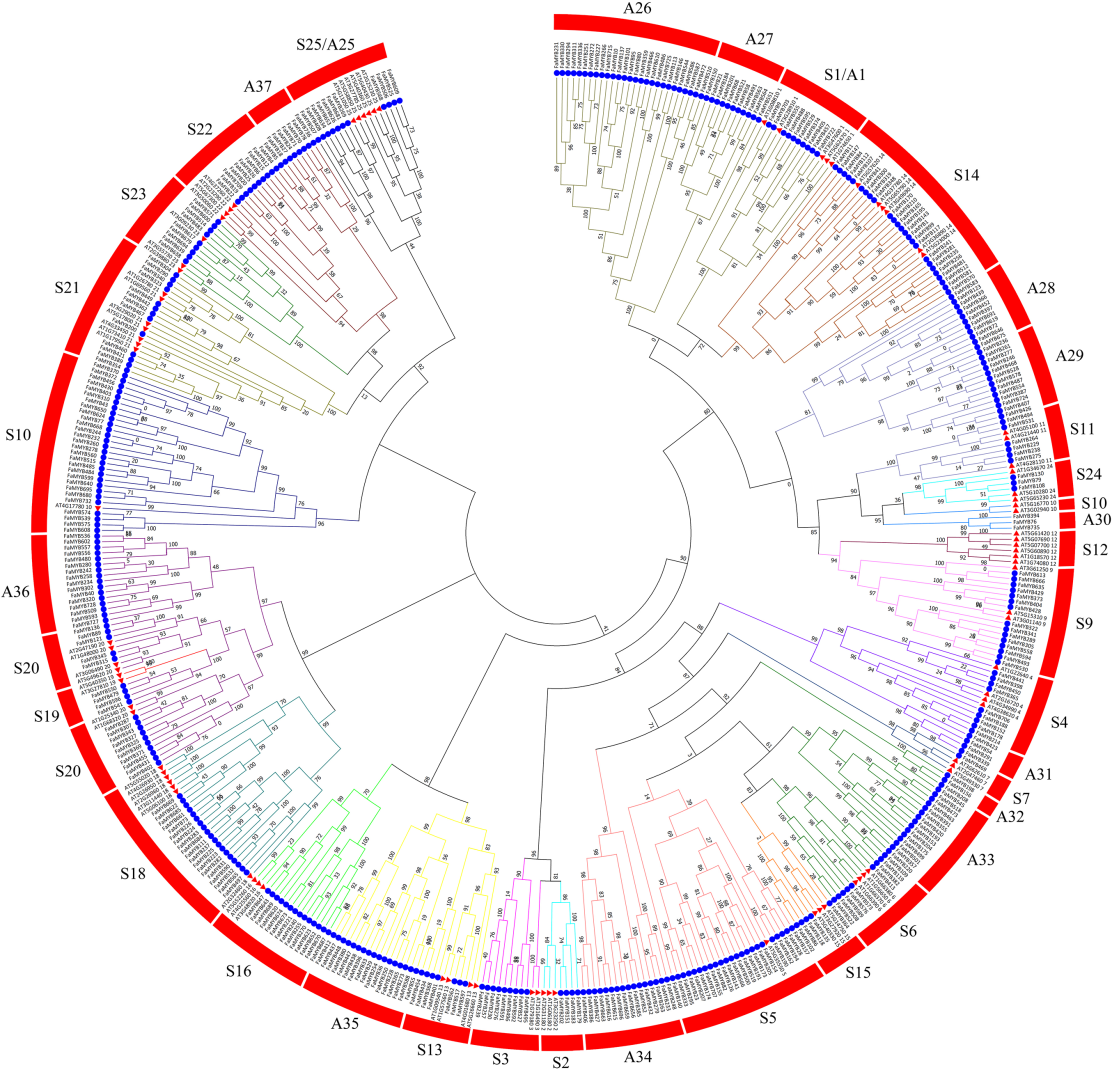
Phylogenetic relationships of R2R3-MYB proteins between *F.* × *ananassa* and *Arabidopsis*. The circles and triangles represented genes from *F.* × *ananassa* and *Arabidopsis*, respectively. All 37 subfamilies of R2R3-MYBs were well separated in different clades and represented by different colors. The ML phylogenetic tree was generated using JTT algorithm with 1,000 bootstrap value *via* MEGA 7.0.

To analyze the phylogenetic relationships and gene functions of the *R2R3-FaMYB* gene family members, a ML tree containing 393 *R2R3-FaMYB* genes and 126 *R2R3-AtMYB*s was constructed using Mega 7.0 software. The 393 *R2R3-FaMYB* genes can be divided into 37 subfamilies (A1–A37) and are drawn in different colors. The A1–A25 groups corresponded to S1–S25 in *Arabidopsis* including 250 *R2R3-FaMYB* genes (Figure 2). There were 11 specific clades (A26–A37) in *F.* × *ananassa* that did not cluster with *Arabidopsis*, while no strawberry *R2R3-FaMYB*s belonged to the A12 *Arabidopsis* subgroup. The group of MYB genes in the same subclade may have a similar function. The R2R3-MYB gene functions in the S4, S5, S6, and S7 subgroups which are known to be involved in the phenylalanine metabolism pathway, including the regulation of anthocyanin and procyanidin synthesis (Dubos et al., 2010). The data showed that all *R2R3-FaMYBs* along with previously identified coloration related MYBs were clustered into four distinct clades (S4–S7). For example, all subgroup 4 R2R3-MYB members were able to inhibit PA biosynthesis, including AT1G22640 (*AtMYB3*), AT4G38620 (*AtMYB4*), AT2G16720 (*AtMYB7*), and AT4G34990 (*AtMYB32*), suggesting that these 13 *R2R3-FaMYBs* in S4 have the ability to participate the PA biosynthesis; AT1G56650 (*AtMYB75*), AT1G66390 (*AtMYB90*), AT1G66370 (*AtMYB113*), and *AtMYB114* (AT1G66380; subgroup 6) control anthocyanin biosynthesis in vegetative tissues, indicating that the 13 *R2R3-FaMYB* members in S6 are anthocyanin-related MYB proteins. Therefore, a total of 54 *R2R3-MYB* genes in the octoploid strawberry were selected candidate proteins related to the coloration.

### Gene Structural Analysis and Conserved Motif Identification of *R2R3-FaMYB*s

Genetic structural analysis is helpful for a better understanding of a gene’s function and evolution. The intronic numbers of the *R2R3-FaMYB* genes varied from 0 to 27 with an average of 2.55; of these, genes with two introns accounted for about 54.1%. There were 16 genes (about 4%) that lacked intronic structures, including *FaMYB4*, *FaMYB5*, *FaMYB6*, and *FaMYB12* (Supplementary Table S2). Most *R2R3-FaMYB* genes contained similar exon-intron distributions of three exons and two introns, accounting for 54.2%. There were also 81 *R2R3-FaMYB*s with two exons and one intron, accounting for 20.6%. Furthermore, genes that exhibited similar exon-intron structures tended to cluster in the same subgroups on the phylogenetic tree, particularly in terms of the number of introns. For example, most genes lacking introns were clustered into the A13 and A21 subgroups. However, some exceptions were also observed. For example, the number of introns in the A26 subgroup ranged from 5 to 8. A13 may have a pleiotropic role, such as influencing lignin deposition, mucilage production and stomatal aperture, according to the known functions of four members in *Arabidopsis thaliana* including AT1G57560 (*AtMYB50*), AT4G01680 (*AtMYB55*), AT1G09540 (*AtMYB61*), and AT5G26660 (*AtMYB86*; Newman et al., 2004). Similarly, the *AtMYBs* in subgroup 21 are proposed to regulate lignin, xylan and cellulose biosynthesis (Dubos et al., 2010). Therefore, the *R2R3-FaMYB*s in A21 may participate the lignin, xylan, and cellulose biosynthesis. According to the BLASTX in NCBI, the members in A26 may participate the phloem parenchyma development and balance between nutrient stress response and immune regulation (Motte and Beeckman, 2017).

The number of amino acids in each conserved motif ranged from 11 (Motif 6) to 50 (Motifs 8, 9, and 13; Supplementary Table S3). Most *R2R3-FaMYB* genes contain Motif 3, with only one gene (*FaMYB725*) lacking the motif, and Motif 13 was seen in only a few genes (2.04%). Motifs 1, 2, 3, and 4 were usually found together in the majority of the R2R3-FaMYB proteins. In general, gene members with similar functions in the same subgroup were likely to exhibit similar motif compositions, but there were significant differences among different subgroups. For example, subgroups 4, 5, 6, and 7 were related to anthocyanin synthesis. All proteins in subgroup 4 possessed Motifs 5 and 8, suggesting that these motifs may be related to the inhibition of anthocyanin synthesis, while all subgroup 6 members contained Motifs 7 and 9, suggesting they may be related to the promotion of anthocyanin synthesis.

### Promoter Regions of *R2R3-FaMYB* Genes

A total of 393 *R2R3-FaMYB* genes were extracted from the upstream 2000bp nucleotide sequences for the prediction of various cis-acting regulatory elements. There were significant differences in the cis-elements of the *R2R3-FaMYB* genes, 12 motifs related to plant development and 13 related to stress response were analyzed (Supplementary Table S4). The predicted data showed that the most common anaerobic induction elements were found in 316 *R2R3-FaMYB* gene promoters, followed by 289 light responsive elements, 284 ABA response elements, and 252 MeJA response elements. We also found some transcription factors were also predicted to bind the *R2R3-FaMYB* promoter regions, such as C2H2, MYB, Dof, NAC, and WRKY, suggesting that these *R2R3-FaMYB* genes may be cross-regulated by other proteins.

### Chromosomal Distribution and Collinearity Analysis of Duplicated *R2R3-MYBs* in *F.* × *ananassa*

**Figure 3:**
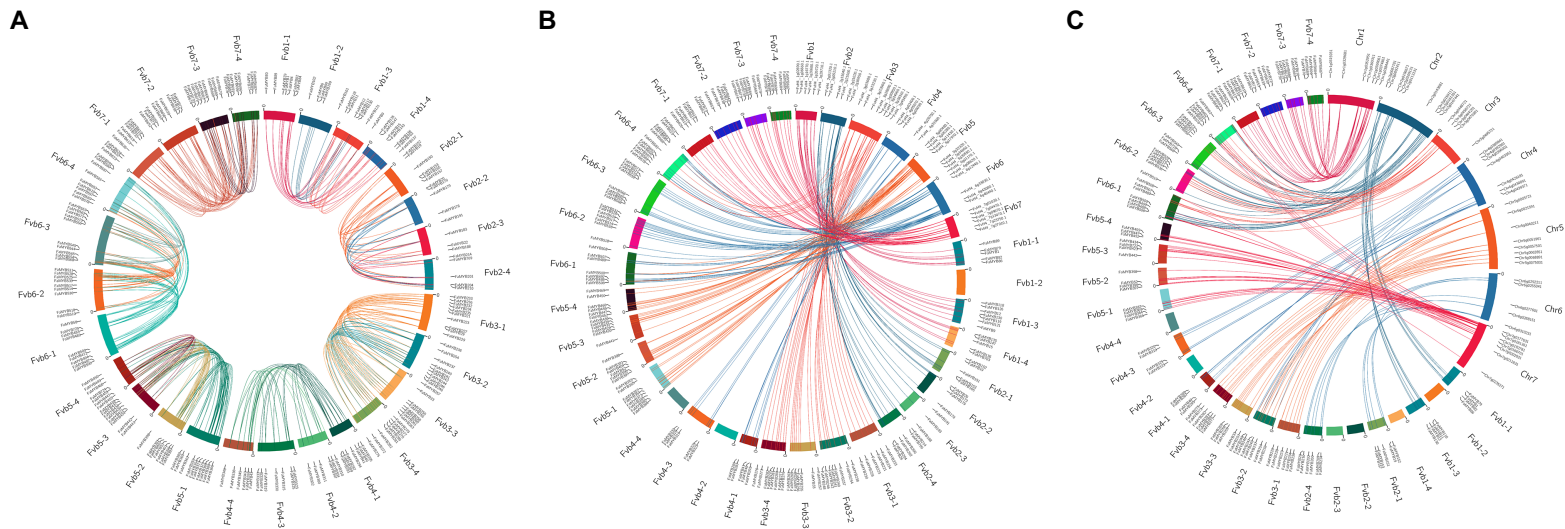
Collinearity analyses of *R2R3-MYB* genes in the *F.* × *ananassa* genome, and among *F.* × *ananassa*, *F. vesca*, and *R. chinensis* genomes. **(A)** Collinearity analysis of R2R3-FaMYBs in *F.* × *ananassa*; **(B)** interspecific collinearity analysis of R2R3-MYBs between *F.* × *ananassa* and *F. vesca*; **(C)** interspecific collinearity analysis of R2R3-MYBs between *F.* × *ananassa* and *R. chinensis*. Outer boxes represented chromosome numbers. Colored lines in boxes indicated the location of *R2R3-MYB* genes in each chromosome. Gene pairs with a syntenic relationship are joined by colored lines.

The chromosomal location analysis revealed that the *R2R3-FaMYB* genes in *F.* × *ananassa* were unevenly distributed over all 28 chromosomes. The density of the genes was highest on chromosome Fvb6-1 with 27 genes, and the lowest on chromosomes Fvb2-2, Fvb4-1, Fvb4-2, and Fvb4-4 with eight genes each (Supplementary Figure S1). Most of the *R2R3-FaMYB* genes were located in the autosomal regions at both ends of the chromosome. There were 40 syntenic gene blocks of *R2R3-FaMYB* genes among the octoploid strawberry chromosomes, and 82.95% of the genes belonged to homologous and collinear genes (Figure 3A; Supplementary Table S5). Based on a genome-wide analysis of gene duplications, a total of 409 pairs of *R2R3-FaMYB* genes showing segmental or tandem duplication were identified, of which only 28 pairs (6.85%) duplicated tandemly into chromosomes, suggesting that segmental duplications may be more beneficial to the expansion of *R2R3-FaMYBs* in the octoploid strawberry.

The comparison of interspecific synteny among *F.* × *ananassa*, *F. vesca*, and *R. chinensis* was also analyzed to further explore the evolution of *R2R3-FaMYB* genes (Figures 3B,C). The number of orthologous gene pairs between *F.* × *ananassa* and *F. vesca*, and between *F.* × *ananassa* and *R. chinensis* was 313 and 266, respectively. Numerous close orthologous relatives of *R2R3-MYBs* were identified in the comparisons with *F.* × *ananassa* and *F. vesca*. The driving force behind the duplication of 409 duplicated gene pairs in *F.* × *ananassa* was analyzed by calculating the Ka and Ks ratio (Supplementary Table S6). The Ka/Ks values of most *R2R3-FaMYB* genes were less than 1, indicating that the evolution of these genes was influenced by purifying selection with limited functional divergence after the duplication events. In contrast, 27 duplications showed Ka/Ks ratios greater than 1, suggesting that these were under positive selection. To further understand the interspecific divergence of the *R2R3-MYB* genes after polyploidization in *F.* × *ananassa*.

### Analysis of the Transcriptome Data and Differential *R2R3-MYB*s

**Figure 4:**
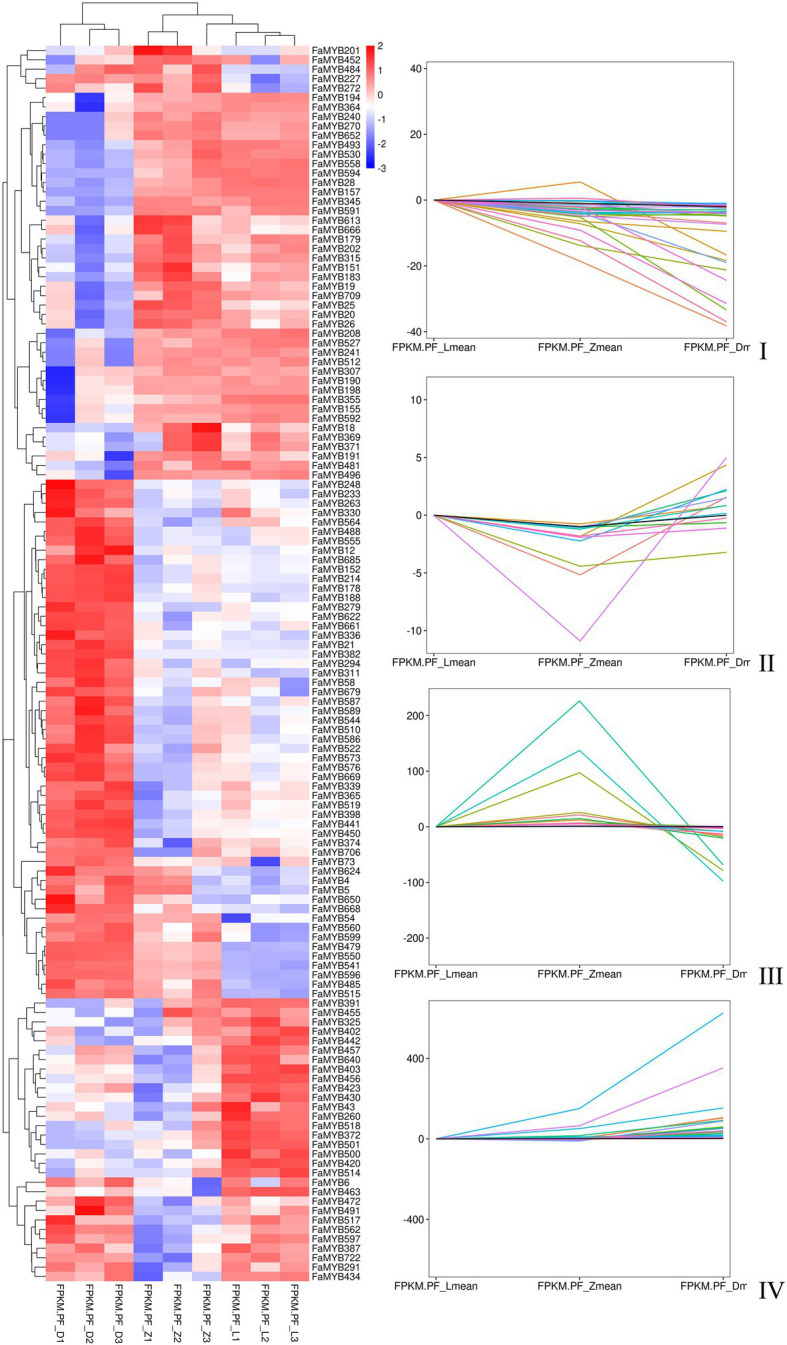
The heat map and trend analysis of 131 differentially expressed *MYB* genes in three flower developmental stages of the pink-flowered strawberry using RNA-seq.

**Figure 5:**
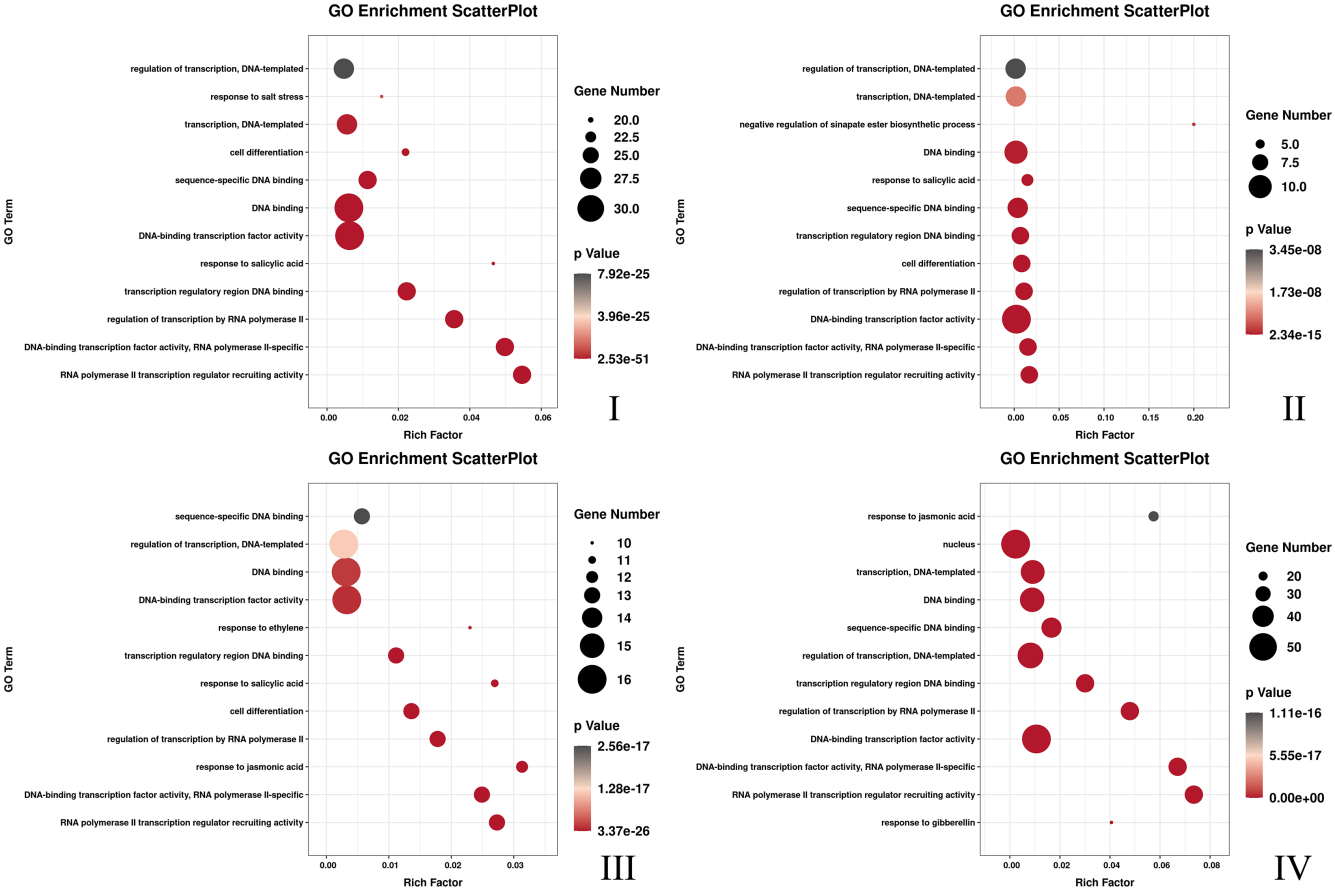
The top 12 GO pathway with the most significant enrichment of each different DE MYBs expression trend. The I, II, III, and IV was corresponding to the Figure 4.

Three flower developmental stages in which petal colors showed marked changes from colorless to dark were used for transcriptome profiling. After removing low-quality reads, a total of 68.68GB high-quality clean reads were obtained from nine samples, with an average of 7.63GB per sample (Supplementary Table S7). The clean reads (91.65% on average) were mapped to the *F.* × *ananassa* reference genome with a Q20 of 99.56% and consistent GC content of 46%. Compared to the genome-wide analysis of the *R2R3-MYB* gene family, it was found 131 MYBs were differentially expressed in transcriptome data. All these differentially expressed genes (DEGs) exhibited differential expressions across petal development of pink-flowered strawberry. The trend analysis showed that these different R2R3-MYBs could have four different expression trends (Figure 4). The GO enrichment analysis was conducted on the differential genes under each trend, and it was found that they were involved in a variety of biological processes, and most of them were likely to perform similar functions, such as DNA-binding transcription factor activity and DNA binding. However, there were some differences under different trends, such as the some DE R2R3-MYBs in trend I response to salt stress; and some response to salicylic acid in trend II (Figure 5). On the other hand, a single phylogenetic tree construction showed that the 131 MYBs could be divided into 13 subgroups (Supplementary Figure S2). By analyzing these with *Arabidopsis thaliana*, the possible functions of these DE MYBs were further determined.

### Identification of Differentially Expressed *R2R3-MYBs* Related to Petal Coloration in the Pink-Flowered Strawberry

**Figure 6:**
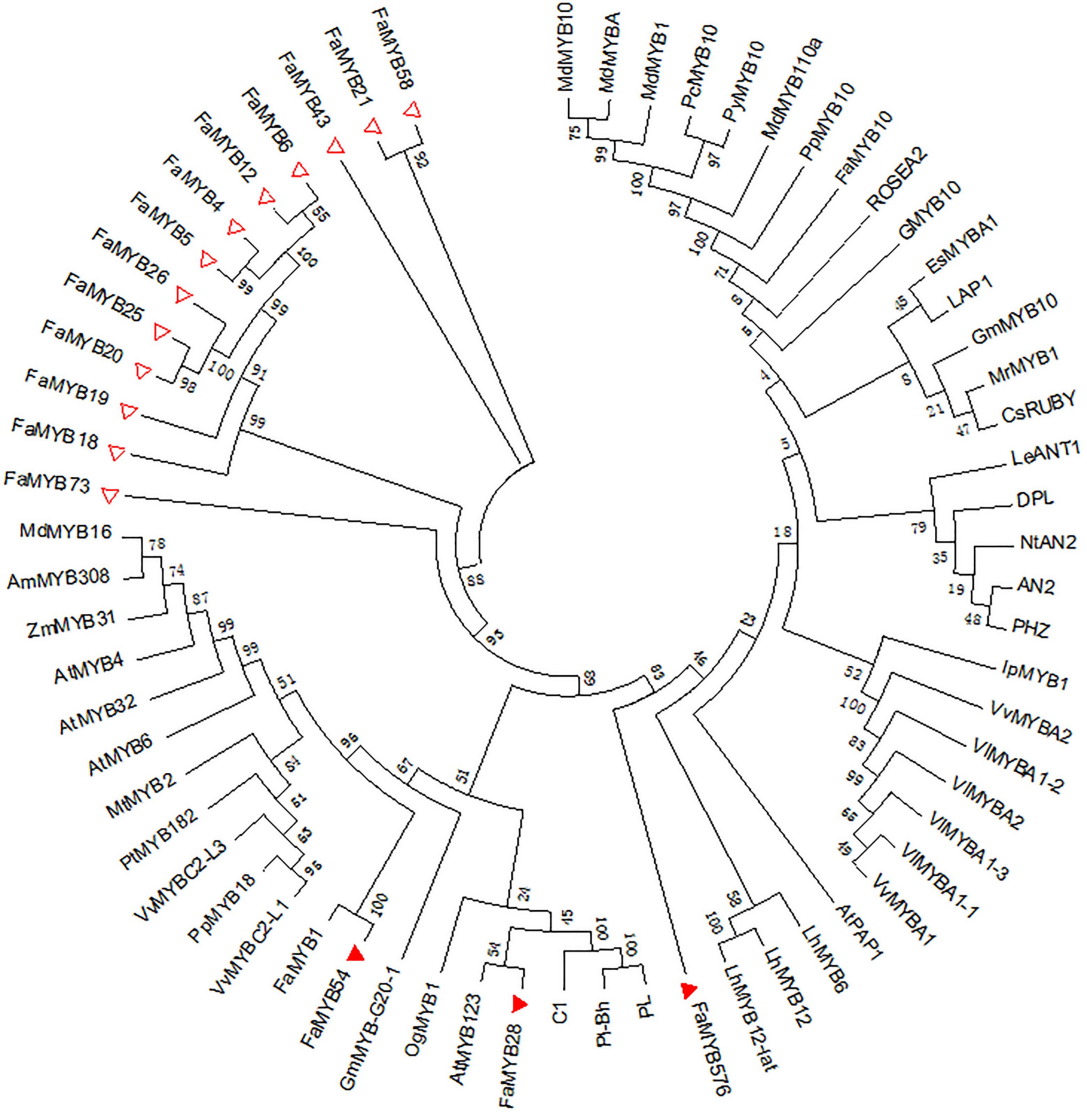
Phylogenetic analysis of the differentially expressed *R2R3-FaMYB* genes in pink-flowered strawberry and other anthocyanin-related R2R3-MYBs. Triangles represent differentially expressed R2R3-FaMYBs in pink-flowered strawberry. The amino acid sequences were retrieved from GenBank databases. Anthocyanin-related MYBs: AtMYB4 (NP_195574), AtMYB123 (NP_198405), AtMYB32 (NP_195225), AtMYBPAP1 (NP_176057), AtMYB6 (NP_192684.1), MdMYB1 (HQ259417), MdMYB10 (EU518249), MdMYB110a (JN711473), MdMYBA (AB279598), MdMYB16 (HM122617.1), MrMYB1 (ADG21957), GMYB10 (CAD87010), VvMYBC2-L1 (AFX64995), VlMYBA1-1 (BAC07537), VlMYBA1-2 (BAC07539), VlMYBA1-3 (BAG55463), VlMYBA2 (BAC07540), VvMYBA1 (BAD18977), VvMYBA2 (BAD18978), VvMYBC2-L3 (KM046932.1), EsMYBA1 (AGT39059), IpMYB1 (AB232769.1), LAP1 (ACN79541), LhMYB6 (BAJ05399), LhMYB12 (BAJ05398), LhMYB12-Lat (BAO04194), C1 (AAA33482), PL (AAA19820), PL-BH (AAA33492), GmMYB10 (ACM62751), PpMYB10 (ABX79945), CsRUBY (AFB73913), OgMYB1 (ABS58501), PcMYB10 (ABX71487), PyMYB10 (ADN26574), FaMYB1 (AAK84064), FaMYB10 (ABX79947), AN2 (AAF66727), DPL (ADW94950), PHZ (ADW94951), ROSEA2 (DQ275530), GmMYB-G20-1 (BAK24100), NtAN2 (ACO52470), LeANT1 (AAQ55181), PpMYB18 (KT159234.1), PtMYB182 (AJI76863.1), MtMYB2 (AES99346.1), AmMYB308 (CAA75691.1), and ZmMYB31 (NP_001105949).

Compared with the 131 different MYBs, 16 of the 54 *R2R3-MYB* genes related to the coloration in the octoploid strawberry were DEGs in the three flower developmental stages, which may regulate the petal coloration in pink-flowered strawberry. An ML tree was constructed by MEGA 7.0 of the 16 *R2R3-FaMYB* DEGs and other *R2R3-MYBs* related to anthocyanin synthesis in different species, including *MdMYB1*, *VlMYBA1-1*, and *PhMYB27* (Figure 6). This showed three *R2R3-FaMYB* DEGs, *FaMYB28*, *FaMYB54*, and *FaMYB576*, that appeared to be related to the anthocyanin synthesis in petal coloration in the pink-flowered strawberry. As shown in Figure 6, *FaMYB1* was the closest homologue with *FaMYB54*, suggesting that *FaMYB54* may negatively regulates the anthocyanin synthesis (Aharoni et al., 2001); *AtMYB123* was the closest homologue with *FaMYB28*, indicating *FaMYB28* may activate the LBGs of the biosynthesis of anthocyanins and PAs (Dubos et al., 2010); *FaMYB576* has closer homologues with other positive regulators dedicated to the anthocyanin synthesis, such as *LhMYB12*, *LhMYB6*, and *AtPAP1* (Yamagishi et al., 2010; Qiu et al., 2014).

**Figure 7:**
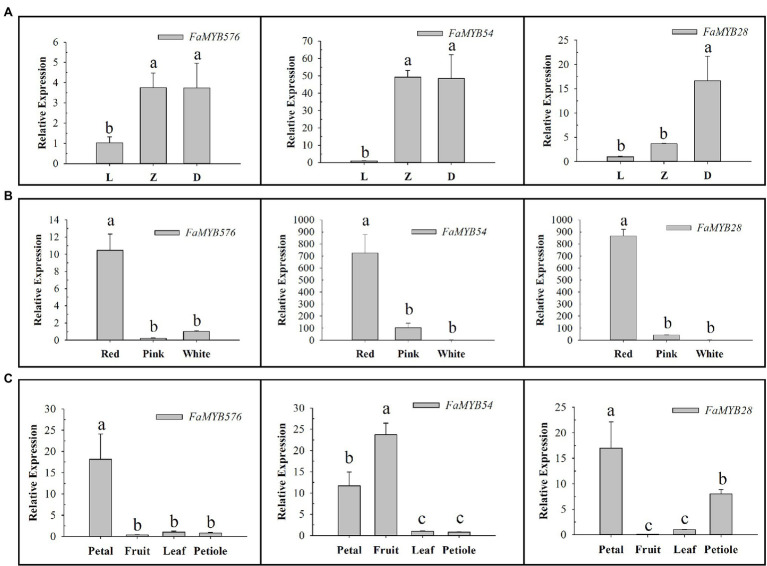
Expression patterns of three anthocyanin-related *R2R3-FaMYB* genes in pink-flowered strawberry. **(A)** Expression patterns of three anthocyanin-related *R2R3-FaMYB* genes in different flower developmental stages (L: Bud stage; Z: Beginning coloration stage; D: Big bud stage). **(B)** Expression patterns of three anthocyanin-related *R2R3-FaMYB* genes in different flower colors. **(C)** Expression patterns of three anthocyanin-related *R2R3-FaMYB* genes in different tissues. Error bars indicate the mean±SE of three independent replicates. Different lowercase letters (a, b, and c) are significantly different (*p*<0.05).

The qRT-PCR results showed that the expression of these three genes increased gradually with the development of flower buds, among which *FaMYB576* and *FaMYB54* showed no significant difference in Z and D stages (Figure 7). Moreover, their expression levels increased gradually with the deepening of color, and the highest expression level was found in red color, suggesting that they may be involved in regulating the anthocyanin accumulation in flower petals of pink-flowered strawberry. Only *FaMYB576* was specifically expressed in petals, which further indicated that *FaMYB576* may regulate the anthocyanin accumulation in pink-flowered strawberry. Meanwhile, *FaMYB54* and *FaMYB28* were also expressed in fruits and petioles, suggesting that *FaMYB54* and *FaMYB28* may be involved in the synthesis of anthocyanin in fruits and petioles of pink-flowered strawberry.

## Discussion

There are currently 24 known species in the *Fragaria* genus with ploidy ranging from diploid (2*n*=2*x*=14) to decaploid (2*n*=10*x*=70), of which the octoploid strawberry (*F*. × *ananassa*) is cultivated commercially around the world (Lei et al., 2016). The genome sequencing of the cultivated octoploid strawberry was completed in 2019 (Edger et al., 2019), providing a useful tool for genome-wide analysis of the *R2R3-FaMYB* gene family. To date, there is no full analysis of the *R2R3-FaMYB* gene family and most functions remain unclear. In this study, we identified 393 *R2R3-FaMYB* genes from the octoploid strawberry genome. This number of *R2R3-MYBs* is significantly different from the numbers in other species, such as 244 in soybeans (Aoyagi et al., 2014), 192 in the poplar (Wilkins et al., 2009), 256 in the Chinese cabbage (Wang et al., 2015), and 205 in cotton (Huang et al., 2019). This study offers new insights for future investigators to identify functional differences in the *R2R3-FaMYB* family genes.

Gene duplication is a major factor responsible for the expansion of gene families and the generation of new genes. Gene duplication includes whole-genome and tandem duplication as well as segmental events (Freeling, 2009; Fang et al., 2012). The size of the octoploid strawberry genome with an estimated genome size of 813.4Mb presents challenges for assembly, compared to the smaller genome of *F. vesca* at 240Mb (Shulaev et al., 2011; Edger et al., 2019). Numerous close orthologous relatives of 85 *R2R3-FvMYB* genes were identified between *F. vesca* and *F.* × *ananassa*. Among these, 25 corresponded to four *R2R3-FaMYB* genes. However, 20 *R2R3-FvMYB* genes were lost in *F.* × *ananassa*. In a few cases, *R2R3-MYB* genes were lost, although this only accounted for a small part of the whole genome, suggesting that the deletion of these genes was not deleterious. Most of *R2R3-FaMYB* gene members underwent purifying selection and were evolutionarily highly conserved, shown by the Ka/Ks analysis. However, a number of *R2R3-FaMYBs* were positively selected, indicating that new gene functions might have been acquired. There was a large amount of collinearity between *F.* × *ananassa* and *F. vesca* and between *F.* × *ananassa* and *R. chinese* in the same Rosaceae family, shown by interspecific microsynteny analysis, which indicated gene duplication at the chromosomal level.

Previous studies have indicated that *R2R3-MYB* genes play key roles in the anthocyanin biosynthesis in various plants, such as *PbMYB10* and *PbMYB114* in pear (Zhai et al., 2016; Yao et al., 2017), *MdMYB1*, *MdMYB10*, and *MdMYB110a* in apple (Espley et al., 2007; Chagné et al., 2013; Hu et al., 2016), and *AcMYB75*, *AcMYB110a*, *AcMYB110*, and *AcMYB123* in kiwi fruit (Fraser et al., 2013; Peng et al., 2019; Wang et al., 2019). In the grape hyacinth, it was found that *MaAN2* was co-expressed with bHLH protein and positively regulated the expression of structural genes in the late stage of the anthocyanin biosynthesis pathway (Chen et al., 2017). *PpMYB18*, a peach *R2R3-MYB* repressor, negatively regulate the accumulation of anthocyanins and proanthocyanidins in peach fruit (Zhou et al., 2018). Fruits of the genus *Fragaria* vary greatly in color, ranging from white to dark red. The concentration and distribution of anthocyanins in fruits also vary widely. The R2R3 type *MYB10* gene is considered a major activator of the anthocyanin pathway in strawberry fruit. Castillejo et al. (2020) identified the *FaMYB10-2* gene, one of three *MYB10* homologs, as the determinant of the natural variation in fruit color in the octoploid strawberry.

The pink-flowered strawberry, derived from the intergeneric hybridization (*Fragaria* × *Potentilla*), increased the strawberry’s ornamental value (Mabberley, 2002). The candidate genes identified in genome-wide co-localization analysis require further investigation to confirm the association between their expressions and flower colors in the pink-flowered strawberry. Different petal colors in different flower developmental stages were used to identify putative *R2R3-FaMYBs via* RNA-seq. In this study, we identified three *R2R3-FaMYBs* that might be involved in the anthocyanin biosynthetic pathway by constructing a phylogenetic tree with *R2R3-MYBs* of known function. The expression levels of *FaMYB576*, *FaMYB28*, and *FaMYB54* in red petals were found to be higher than in pink and white petals. Using NCBI BLASTx, we speculate that *FaMYB576*, *FvMYB90*, and *RmMYBAN2* may have similar gene functions, that *FaMYB28*, *FaMYB9*, and *AtMYB123* may have similar functions, as may *FaMYB54* and *FaMYB1*. In the red Chinese cabbage, transcriptome analysis showed that *BrMYB90* was strongly expressed in the anthocyanin biosynthetic pathway (Rameneni et al., 2020). In *Eutrema salsugineum*, *EsMYB90* was identified as a regulator of anthocyanin biosynthesis and could enhance anthocyanin accumulation in various organs *via* ectopic expression in tobacco and *Arabidopsis* (Qi et al., 2020). *PalMYB90* is highly expressed in *Populus*, and was found to regulate key flavonoid pathways to elevate anthocyanin levels in transgenic plants (Bai et al., 2019). These results suggest that *FaMYB576* should be considered an important candidate gene involved in the regulation of anthocyanin biosynthesis. These data suggest the potential for *FaMYB576* in influencing anthocyanin biosynthesis in the petals of the pink-flowered strawberry, affecting strawberry petal coloration.

## Conclusion

The current study presents the first detailed genome-wide analysis of the *R2R3-FaMYB* genes in the octoploid strawberry. A total of 393 *R2R3-FaMYB* genes were identified and investigated in terms of their protein sequence properties, phylogenetic relationships, gene structures, motifs, and gene duplications. These *R2R3-FaMYBs* were divided into 36 subfamilies based on conserved domain and phylogenetic analyses. Collinearity analysis showed that gene duplication events contributed to the expansion of the *R2R3-MYB* family in *F.* × *ananassa*. There were 131 differentially expressed *MYB* genes identified in three flower developmental stages of the pink-flowered strawberry using RNA-seq, of which three were found to regulate anthocyanin synthesis responsible for petal coloration. These results provide insights into the roles of novel *R2R3-MYB* transcription factors in anthocyanin synthesis and a foundation for further functional analysis to characterize the roles of *R2R3-MYBs* in *F*. × *ananassa* and to develop new approaches to improve the ornamental features of the strawberry.

## Data Availability Statement

The original contributions presented in the study are publicly available. This data can be found here: The sequencing data can be found in NCBI Gene Expression Omnibus (GEO) database (https://www.ncbi.nlm.nih.gov/geo/) under the accession number of GSE125777.

## Author Contributions

JL and JW conceived the study and drafted the manuscript. MW and TZ analyzed the data. JZ and YZ performed the experiments. LX and JL revised the manuscript and provided guidance on the whole study. All authors contributed to the article and approved the submitted version.

## Conflict of Interest

TZ was employed by the company Genepioneer Biotechnologies Co. Ltd.

The remaining authors declare that the research was conducted in the absence of any commercial or financial relationships that could be construed as a potential conflict of interest.

## Publisher’s Note

All claims expressed in this article are solely those of the authors and do not necessarily represent those of their affiliated organizations, or those of the publisher, the editors and the reviewers. Any product that may be evaluated in this article, or claim that may be made by its manufacturer, is not guaranteed or endorsed by the publisher.

## Footnotes

^1^ https://www.rosaceae.org/species/fragaria_x_ananassa/genome_v1.0.a1

^2^ http://bioinformatics.psb.ugent.be/webtools/plantcare/html/

^3^ https://ccb.jhu.edu/software/stringtie

## Supplementary Material

Supplementary Figure S1 Localization and distribution of the identified *R2R3-FaMYB* genes on 28 chromosomes of *F.* × *ananassa*. The numbers at the top of each bar represented different chromosomes. The gene names on each chromosome corresponded to the approximate locations of each R2R3-FaMYB gene.

Supplementary Figure S2 A single ML phylogenetic tree of 131 different *R2R3-FaMYB* genes using MEGA7.0. The reliability of the predicted tree was tested using bootstrapping with 1,000 replicates and JTT model.
