# Supplementary material for: Genome-Wide Analysis of the R2R3-MYB Gene Family in Fragaria × ananassa and Its Function Identification During Anthocyanins Biosynthesis in Pink-Flowered Strawberry
Source: Front Plant Sci. 2021 Aug 30;12:702160. doi: 10.3389/fpls.2021.702160 (PMC8435842; doi:10.3389/fpls.2021.702160)
Supplement: Supplementary file 1 [file Data_Sheet_1.zip › Supplementary Material/Table S1 The primers used for qRT-PCR analysis.docx]

Table S1 The primers used for qRT-PCR analysis

| gene | orientation | sequence |
| --- | --- | --- |
| FaMYB576 | Forward | TGGTGATTTCGGGATGGTAGACC |
|  | Reverse | GTCATCCCACTCCCATTTGTTGT |
| FaMYB28 | Forward | TGGCCGAACAGACAATGAAATCA |
|  | Reverse | TGATTTGGTGGTTTGTGGTGAGT |
| FaMYB54 | Forward | AGGGTTGCGTCGTTGTGGTA |
|  | Reverse | TTCCAGGCAGTCTTCCAGCT |
| FaDBP  (reference) | Forward | TTGGCAGCGGGACTTTACC |
|  | Reverse | CGGTTGTGTGTGACGCTGTCAT |
