# Supplementary material for: Genome-Wide Analysis of the R2R3-MYB Gene Family in Fragaria × ananassa and Its Function Identification During Anthocyanins Biosynthesis in Pink-Flowered Strawberry
Source: Front Plant Sci. 2021 Aug 30;12:702160. doi: 10.3389/fpls.2021.702160 (PMC8435842; doi:10.3389/fpls.2021.702160)
Supplement: Supplementary file 1 [file Data_Sheet_1.zip › Supplementary Material/Table S3 Conserved motifs and predicted structures of R2R3-FaMYB genes in F. ananassa.docx]

Table S3 Conserved motifs and predicted structures of *R2R3-FaMYB* genes in *F*. × *ananassa*

| Number | E-value | Locus | Size | Predicted structure |
| --- | --- | --- | --- | --- |
| motif1 | 4.2E-7155 | 27564 | 41 | 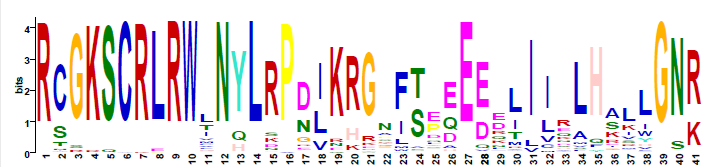 |
| motif2 | 8.6E-5075 | 20140 | 29 | 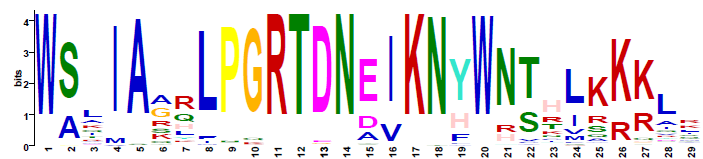 |
| motif3 | 8.1E-1959 | 9719 | 15 | 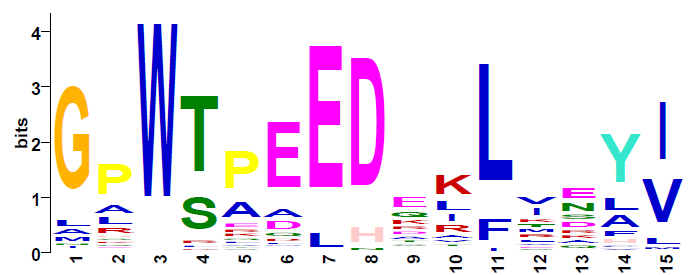 |
| motif4 | 4.0E-1414 | 7445 | 15 | 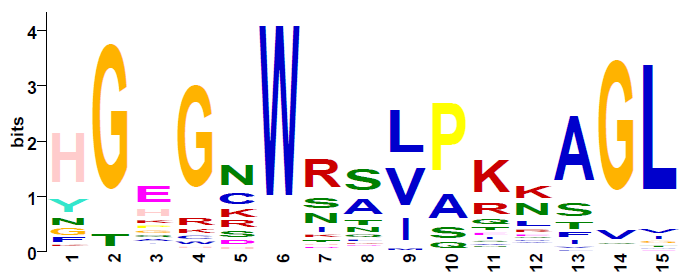 |
| motif5 | 6.2E-719 | 3456 | 41 | 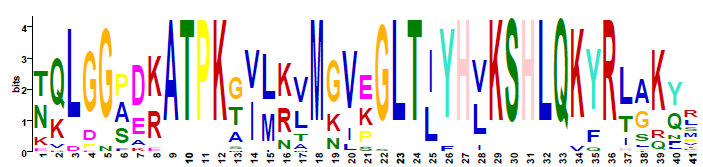 |
| motif6 | 4.5E-661 | 3706 | 11 | 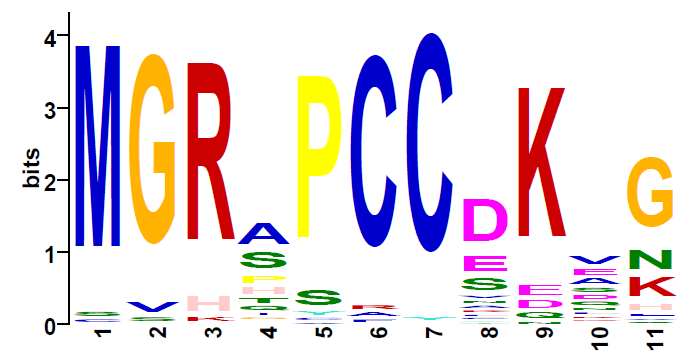 |
| motif7 | 2.6E-567 | 2939 | 35 | 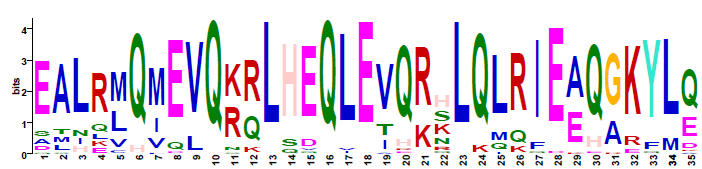 |
| motif8 | 9.1E-507 | 2410 | 50 | 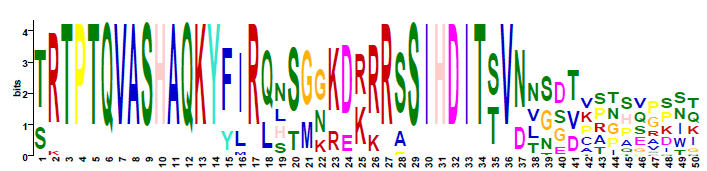 |
| motif9 | 2.8E-479 | 2318 | 50 | 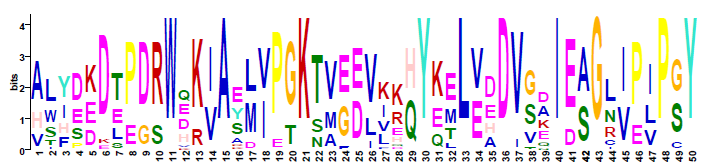 |
| motif10 | 1.4E-305 | 2465 | 15 | 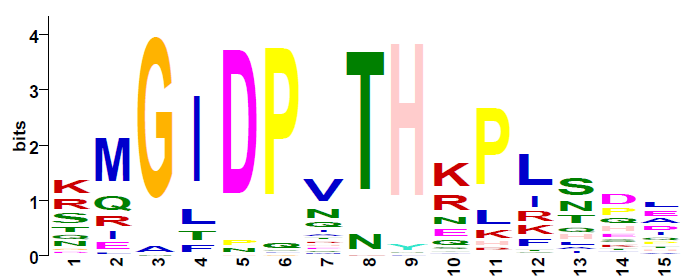 |
| motif11 | 4.4E-251 | 1205 | 29 | 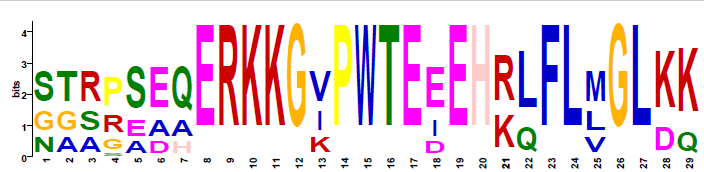 |
| motif12 | 5.2E-199 | 1378 | 15 | 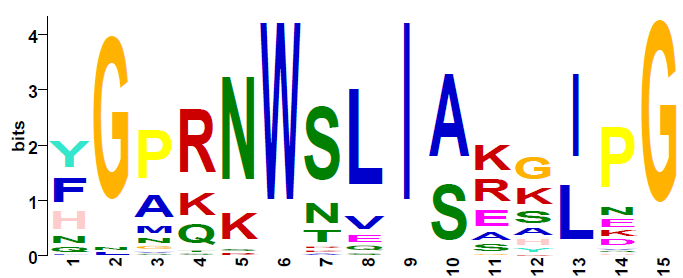 |
| motif13 | 5E-181 | 975 | 50 | 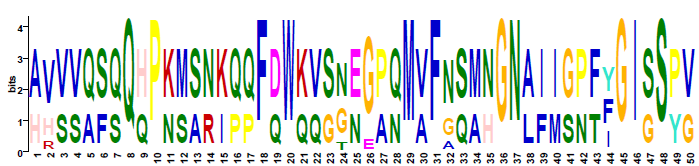 |
| motif14 | 2.3E-200 | 1575 | 41 | 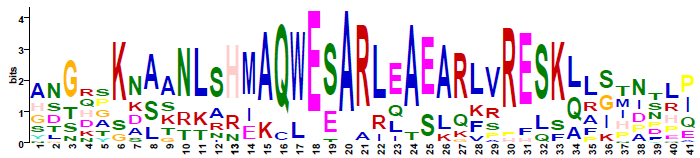 |
| motif15 | 4.1E-171 | 1073 | 29 | 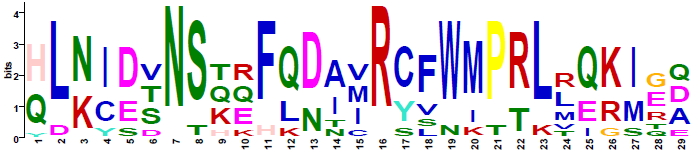 |
