# Supplementary figures and images for: Genome-Wide Analysis of the R2R3-MYB Gene Family in Fragaria × ananassa and Its Function Identification During Anthocyanins Biosynthesis in Pink-Flowered Strawberry
Source: Front Plant Sci. 2021 Aug 30;12:702160. doi: 10.3389/fpls.2021.702160 (PMC8435842; doi:10.3389/fpls.2021.702160)

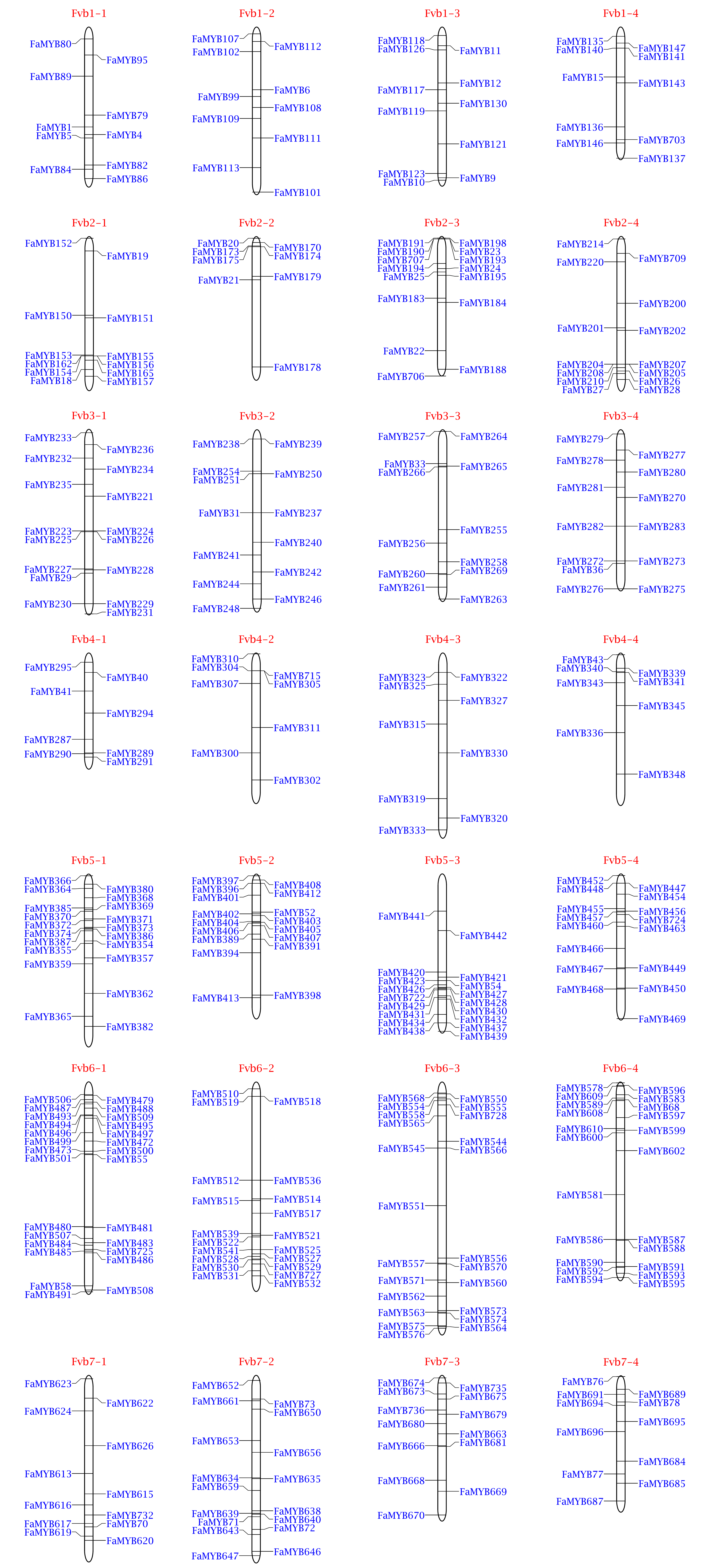

Supplement: Supplementary file 1 [file Data_Sheet_1.zip › Supplementary Material/FIGURE S1 Localization and distribution of the identified R2R3-FaMYB genes on 28 chromosomes of F. ananassa.jpg]

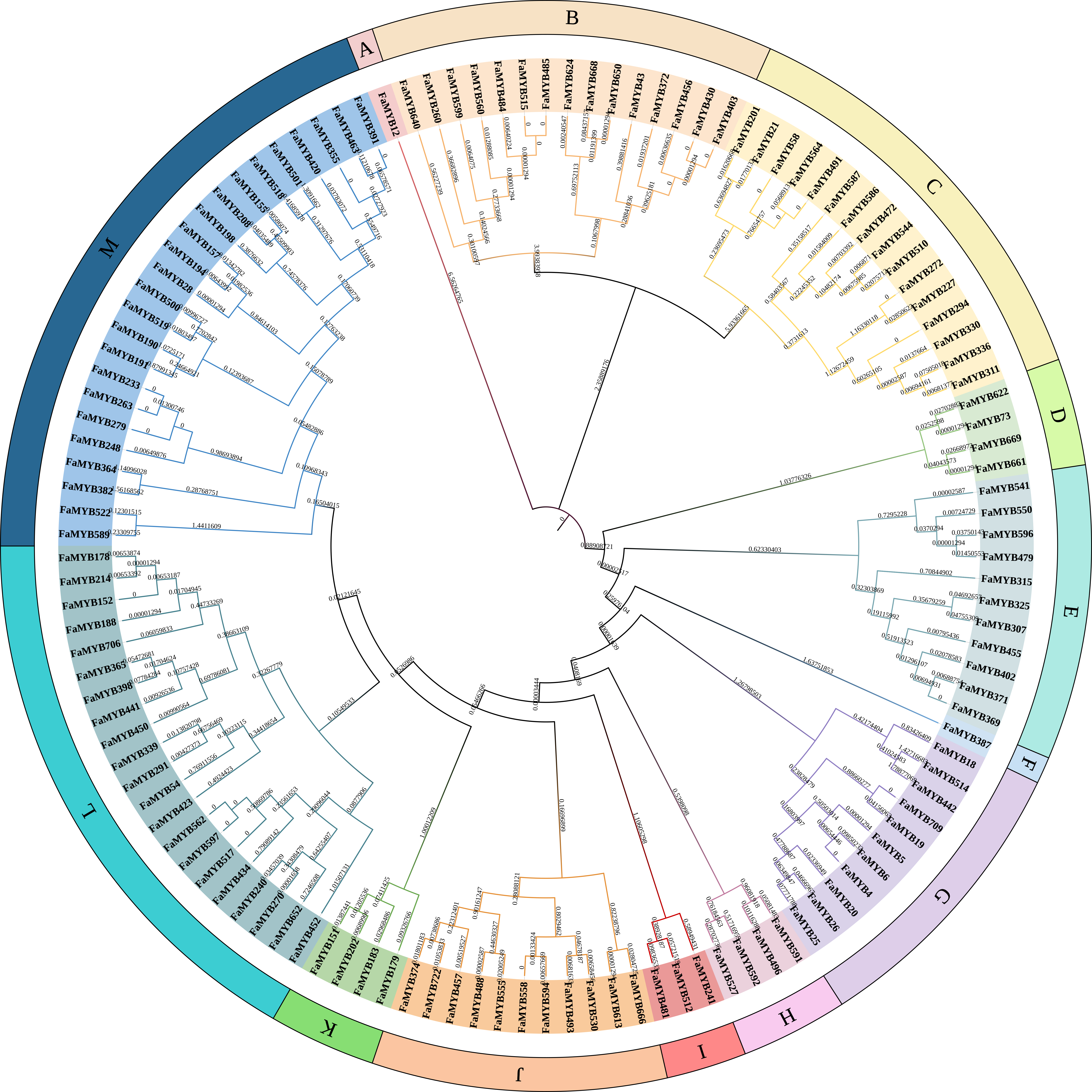

Supplement: Supplementary file 1 [file Data_Sheet_1.zip › Supplementary Material/Figure S2 A single ML phylogenetic tree of 131 different R2R3-FaMYB genes using MEGA7.0.jpg]
